# The red alga *Tsunamia transpacifica* (Stylonematophyceae) from plastic drift shows adaptation to its uncommon habitat in ultrastructure and soluble low molecular weight carbohydrate composition

**DOI:** 10.1007/s00709-021-01674-6

**Published:** 2021-06-25

**Authors:** Andreas Holzinger, Sabrina Obwegeser, Ancuela Andosch, Ulf Karsten, Christina Oppermann, Wolfgang Ruth, Allison van de Meene, Christopher D. Goodman, Ursula Lütz-Meindl, John A. West

**Affiliations:** 1 Department of Botany, Functional Plant Biology, University of Innsbruck, 6020 Innsbruck, Austria; 2 Department of Biosciences, University of Salzburg, 5020 Salzburg, Austria; 3 Institute of Biological Sciences, University of Rostock, Rostock, Germany; 4 Institute of Chemistry, University of Rostock, Rostock, Germany; 5 School of BioSciences, University of Melbourne, Parkville, VIC 3010 Australia

**Keywords:** Cobalt, Digeneaside, Floridoside, Iron, Phosphorus, Red algae

## Abstract

The recently described red alga *Tsunamia transpacifica* (Stylonematophyceae) was previously isolated from plastic drift found at the pacific coast, but the natural habitat remains unknown. Here, we investigate ultrastructural details and the low molecular weight soluble carbohydrate composition to get further insight into the adaptation to this uncommon habitat. By means of high pressure freeze fixation, followed by freeze substitution, we could detect an up to 2-µm-thick cell wall surrounded by a distinct layer of extracellular polymeric substances (EPS), likely responsible for the adhering capacities of *Tsunamia*. The central position of the nucleus and multilobed parietal chloroplast, already observed by light microscopy, could be confirmed. The ultrastructure revealed large electron-dense bodies (EB) in the central cytoplasm, likely resembling degradation products of the chloroplast. Interestingly, these structures contained phosphorous and cobalt, and iron was found in smaller rounded electron-dense bodies by electron energy loss spectroscopy (EELS). Accumulation of these elements suggests a high biosorption activity of *Tsunamia*. Liquid chromatography-mass spectrometry (LC–MS) data showed the presence of two heterosides (floridoside and digeneaside) together with the polyol sorbitol, which are known as organic osmolytes and compatible solutes. Taken together, these are the first observations on ultrastructural details, element storage and accumulation of protective compounds are contributing to our understanding of the ultrastructural and osmotic solute basis for the ability of *Tsunamia* to thrive on plastic surfaces.

## Introduction

The red alga *Tsunamia transpacifica* J.A. West, G.I. Hansen, G.C. Zuccarello et T. Hanyuda (West et al. 2016), was previously detected on a rather uncommon habitat—plastic debris originating from a tsunami resulting from a tremendous earthquake off the pacific coast of Tohoku, Japan, that took place on March 11, 2011. The debris crossed the Pacific Ocean and started to emerge on the Northeastern Pacific shores since 2012, where a conspicuous pink colored red algal crust was isolated in 2015 (West et al. 2016). Many so far unanswered questions came up; how this organism lives remains undetected in its natural habitat and what kind of structural and physiological properties would enable to survive in the changing habitat.

The algal species diversity on such debris becomes increasingly interesting and has been studied, for example, by Hanyuda et al. (2018) who were able to obtain gene sequences from 205 specimens of which 49 species were identified as Japanese tsunami marine debris. Hansen et al. (2018) analyzed 42 heavily fouled debris items and detected many taxa with high invasion potential, as 83% were reproductive, 48% ephemeral, and 75% were opportunistic life forms. The phylogenetic variety contained taxa from the Phaeophyceae (20 species), Ulvophyceae (13 species), and Rhodophyta (16 species) (Hanyuda et al. 2018). The red algal genera contained species from *Bangia*, *Ceramium*, *Chondrus*, *Grateloupia*, *Neodilsea*, *Palmaria*, *Polysiphonia*, *Pyropia*, *Schizymenia*, and the here investigated genus *Tsunamia*. These genera come from different classes like Bangiophyeae (2), Florideophyceae (7), and Stylonematophyceae (1). Recently, another new red algal genus from the Stylonematophyceae, *Viator vitreocola*, has been described on drift glass debris collected in Oregon and Washington (Hansen et al. 2019), suggesting that the substrate is specific for the different genera. All these algal organisms have in common that they exhibit strong adhesive properties and hence stick tightly to the different man-made surfaces.

*Tsunamia transpacifica* grows readily in laboratory culture but very little is known of its general biology. Moreover, the natural habitat for this rare red alga has not been described. *Tsunamia transpacifica* was characterized as a small pulvinate crust of radiating, branched, uniseriate filaments with cells containing a central nucleus and a single, purple to pink, multilobed parietal plastid, lacking a pyrenoid (West et al. 2016). Molecular phylogeny places *T. transpacifica* firmly in the Stylonematophyceae (West et al. 2016), together with 18 other genera from marine, brackish, freshwater, terrestrial soil, and epizoic habitats. The position of *T. transpacifica* and *V. vitreocola* in the phylogenetic tree is fully supported by ML bootstrap values and Bayesian posterior probabilities (Hansen et al. 2019). However, no molecular analyses have been possible so far for other closely related genera (*Colacodictyon*, *Empselium*, *Glauconema*, *Kneuckeria*, *Neevea*, and *Phragmonema*), all provisionally placed in the Stylonematophyceae because none of these have been cultured.

The general characteristics of the class Stylonematophyceae are unicellular, colonial, prostrate, and upright filaments; reproduction by monospores in most genera formed directly from vegetative cells or multiple autospores in *Rhodospora*; sexual reproduction is unverified. Red or blue-green cells, (blue-green cells of *Chroothece* (Pentecost et al. 2013) and *Chroodactylon* have only phycocyanin, phycoerythrin is absent due to a gene deficiency). The cells are uninucleate and contain a single peripheral multilobed plastid or several small plastids with a peripheral encircling thylakoid, either with or without pyrenoids. Golgi bodies are associated with the endoplasmic reticulum. Low molecular weight soluble carbohydrates are mostly represented by digeneaside, sorbitol, and floridoside; floridean starch granules are localized in the cytoplasm, and no pit plugs between cells, cell walls, and extracellular matrix consist of complex polysaccharides.

The ultrastructure of red algae in the class Stylonematophyceae has been studied only in a few taxa grown in laboratory culture. Examples are the unicellular genus *Rhodosorus* (Broadwater and Scott 1994; Ford 1984; Wilson et al. 2002) which showed excellent cellular details with conventional TEM methods. The epiphytic filamentous marine to brackish genera *Purpureofilum*, *Bangiopsis* (West et al. 2005), and *Rhodaphanes* (West et al. 2007) have thick cell walls and extracellular matrices that interfere with fixation but good preservation in spores and young filaments. *Chroothece*, *Chroodactylon*, and *Goniotrichopsis* are colonial/filamentous also with thick walls and extracellular matrices but the ultrastructure of these genera in culture has not yet been investigated. *Rhodospora* (Johansen et al. 2005) is a freshwater coccoid unicell with thick walls that grow well, reproducing numerous autospores in culture. TEM has not been studied, although Pickett-Heaps et al. (2001) investigated cell motility and found it not motile.

With the uncommon habitat of *T. transpacifica*, directly exposed to high irradiation, possibly limited in nutrition, several cell biological questions came up. How can this organism survive attached to the newly formed artificial habitat? We hypothesized (1) that the uncertainty of the habitat requests protection mechanisms against high irradiation on the open ocean and dehydration when drifted ashore, and (2) that certain storage mechanisms must exist that allow this alga to live in open off-shore waters under nutrient limiting conditions. Furthermore, we also hypothesize (3) that the cells must have mechanisms to adhere to the plastic surface and must build up cell walls that support this adhesion and are strong enough to withstand mechanical damage. Therefore, we performed a detailed ultrastructural investigation by transmission electron microscopy, followed by electron energy loss spectroscopy (EELS) measurements to analyze element distribution in previously uncharacterized electron-dense bodies. Moreover, a characterization of low molecular weight soluble carbohydrates was performed by a LC–MS approach to test for osmotic protectants preventing damage under desiccating conditions. With this dataset, we aim to get more insight into the cell biological properties of a rare red alga isolated from an uncommon habitat.

## Material and methods

### Algal material

At the University of Melbourne, *Tsunamia transpacifica* J.A. West, G.I. Hansen, G.C. Zuccarello et T. Hanyuda (culture strain 4874, West et al. (2016)) was cultured in natural seawater (NSW, 30–32 S_A_, ca. 950 mOsmol kg^−1^) with modified Provasoli’s medium (MPM) nutrient enrichment (West et al. 2005), 10:14 h light/dark (LD) cycle, ca 5- to 10-µmol photons m^−2^ s^−1^ cool white LED illumination, in 70 × 50 mm Pyrex glass dishes (with about 80- ml medium), 18–22 °C, stationary, or 70-rpm rotary shaker.

Due to the lack of NSW at the University of Innsbruck, *T. transpacifica* was cultured slightly differently, in 34-g L^-1^ “Morton® Sea Salt” (MSS, 1018 mOsmol kg^−1^), which has the same chemical composition as NSW and supplemented with an identical MPM enrichment, grown either at ~ 6-µmol photons m^−2^ s^−1^ in a shady laboratory space at 20 °C, 10:14 h LD, or at 14 °C, 16:8 h LD, ~ 30 µmol photons m^−2^ s^−1^ illuminated by OSRAM L36W77 Fluora and OSRAM L36W 840 Lumlux “cool white” lamps mounted outside a glass window in an custom built incubation chamber.

### Light and confocal laser scanning microscopy

Light microscopy of *T. transpacifica* was performed with a Zeiss Axiovert 200 M under the control of Zeiss AxioVision software (release 4.7) and images were captured with a Zeiss AxioCam HRm Rev.3 camera or with a Zeiss GFL bright field microscope (Zeiss Australia, Sydney, Australia) and Canon G3 camera (https://www.canon.com.au/). Confocal laser scanning micrographs were obtained by a Zeiss Pascal LSM5 under control of ZEN 2009 SP2 software (cells grown at the University of Innsbruck) or a Nikon C2 using NIS Elements software (cells grown at the University of Melbourne). For DNA staining, performed to visualize the cell’s nuclei, *Tsunamia* cells were fixed either by (1) immersing in 4% paraformaldehyde fixative (Electron Microscopy Sciences, Hatfield, PA, USA) for 10 min (for details of the protocol, see Hansen et al. 2019) or by (2) microwaving in distilled water in a plastic petri dish for 3 successive 3- to 4-s intervals at 800 W as previously described (Hansen et al. 2019). They were then rinsed in seawater and stained with 1 µg ml^-1^ Hoechst 33342 (Thermo Fisher, Waltham, MA, USA) or 1:10,000 SYBR Green (c) for 5 min. Following staining, the cells were washed in milliQ water, mounted under a 22-mm coverslip in AntiFade DAKO fluorescent mounting media (Agilent, Santa Clara, CA, USA). Stained cells were imaged with a Nikon C2 confocal microscope (Japan, the Biological Optical Microscopy Platform, University of Melbourne).

### Transmission electron microscopy

*Tsunamia transpacifica* was prepared for TEM either by a standard chemical fixation protocol, fixed in 2.5% glutaraldehyde in 50 mM cacodylate buffer (pH 6.8) for 1 h, post-fixation in 1% OsO_4_ for 18 h (according to Holzinger et al. 2004), dehydration by increasing ethanol concentration, and embedded in modified Spurr’s low viscosity resin (Sigma-Aldrich, St. Louis, USA). As this method did not give reasonable results, a high pressure freeze (HPF) fixation protocol, according to the methods described by Aichinger and Lütz-Meindl (2005), was applied. *Tsunamia transpacifica* cells grown at the University of Innsbruck were fixed with a LEICA EMPACT high pressure freezer and freeze substituted in a Leica EM AFS FS (Leica Microsystems GmbH, Vienna, Austria) apparatus, in 2% OsO_4_ and 0.05% uranyl acetate in acetone at − 80 °C for 60 h, temperature raised to − 30 °C within 5 h (10 °Ch^-1^), maintained at − 30 °C for 4 h, and temperature raised to 20 °C within 20 h (2.5°C h^-1^). Samples were then embedded in agar low viscosity resin kit (Agar Scientific, Essex, UK). Ultrathin sections (60 nm) were prepared with a Reichert Ultracut S (Leica AG, Vienna, Austria) and post-stained with 2% uranyl acetate (10 min) and Reynold’s lead citrate (2 min), examined with a Zeiss Libra 120 transmission electron microscope (80 kV) and photographed with TRS 2 k SSCCD camera. *Tsunamia transpacifica* cells grown at the University of Melbourne were high pressure frozen similarly by using a Leica EM ICE HPF (Leica Microsystems) following the protocol from Wetherbee et al. (2019). Briefly, the cells were placed in 3-mm carriers without cryoprotectants. Following HPF, the cells were freeze substituted in 2% OsO_4_ in acetone at − 85 °C for 72 h. The samples were then slowly warmed to room temperature over the next 48 h, washed three times in acetone followed by infiltration with Spurr’s resin (ProSciTech). Ultrathin sections (70 nm) were prepared using a Leica UC7 ultramicrotome (Leica Microsystems) and imaged using a Thermo Fisher FEI Tecnai Spirit transmission electron microscope equipped with an Eagle 2 K CCD camera (FEI, Hillsboro, OR, USA).

### Electron energy loss spectroscopy (EELS)

For EELS, 40- to 50-nm-thick sections of *T. transpacifica* were mounted on hexagonal narrow mesh uncoated copper grids. Sections were investigated in a LEO 912 AB TEM (Zeiss, Oberkochen, Germany) with in-column energy filter at 120 kV for EELS (according to the methods described in Andosch et al. 2015). Images were acquired by a TRS Sharpeye dual speed slow scan CCD camera (Tröndle, Mohrenwies, Germany) run by ITEM Software (SIS, Soft Imaging System, Münster, Germany). The magnification for EELS was × 20,000, illumination angles between 1.6 and 2 mrad, and camera exposure time between 5 and 100 s (with 7 integration cycles). The measured area for EELS was defined by using a 100-µm spectrometer entrance aperture with a spectrometer magnification of × 125. From the initial wide range EELS measurements (detecting C, Ca, O, P, Al, Mg, Co, Fe, etc.), we selected the most abundant elements and performed detailed measurements in selected areas. Phosphorus (P) was identified by the K-edge at 2146 eV, cobalt (Co) by the L3-edge at 779 eV, and iron (Fe) determined by the L3-edge at 708 eV. At the University of Melbourne, a Thermo Fisher FEI TF30 transmission electron microscope equipped with a Gatan Quantum 965 energy filter was used for the analysis.

### Low molecular weight soluble carbohydrate (LMWC) analysis

In dry algal samples, each of which 2–3 mg was extracted with 70% aqueous ethanol (v/v) in capped centrifuge tubes at 70 °C in a water bath for 4 h according to Karsten et al. (1991). After centrifugation for 5 min at 5000 g, 700 µl of the supernatant was evaporated to dryness under vacuum (Speed Vac Concentrator SVC 100H, Savant Instruments Inc, Holbrook, NY, USA). Dried extracts were re-dissolved in 700 µl distilled water and vortexed for 30 s. Samples were analyzed with an isocratic Agilent HPLC system (Santa Clara, CA, USA) equipped with a differential refractive index detector. LMWCs were separated and quantified by two high-performance liquid chromatography (HPLC) methods in order to maximize peak identification. Separation of polyols and disaccharides was performed on a Bio-Rad resin-based column (Aminex Fast Carbohydrate Analysis, 100 × 7.8 mm, Bio-Rad Inc, Hercules, CA, USA) using a Phenomenex Carbo-Pb2 + (4 × 3 mm) guard cartridge. LMWCs were eluted with 100% HPLC grade water at a flow rate of 1 ml min^−1^ at 70 °C (modified after Karsten et al. 1991). Separation of heterosides was performed on a Phenomenex resin-based column Rezex ROA-Organic Acid (300 × 7.8 mm) protected with a Phenomenex Carbo-H + (4 × 3 mm) guard cartridge (Phenomenex Inc, Torrance, CA, USA). On the latter column, LMWCs were eluted with 5-mM H_2_SO_4_ at a flow rate of 0.4 ml min^−1^ at 75 °C (modified after Karsten et al. 2005). LMWCs were identified by comparison of retention times with those of the standard compounds prepared as 1-mM aqueous solutions and quantified by peak areas. All concentrations are expressed in micromole per gram dry weight.

Since the used dry algal material was very low, additional liquid chromatography-mass spectrometry measurements were undertaken for substance verification. The analyses were carried out on a Accela high-performance liquid chromatography (HPLC) system comprising of a Accela 1250 pump (Thermo Scientific™), a cooling Accela autosampler, and a Accela column oven with temperature control (Thermo Scientific™). The data were processed with Xcalibur™ Software (version 3.0.63) and visualized with Origin® 2017. Separations were performed on a Rezex™ ROA-Organic Acid H + (8%) column (250 × 4.6 mm, Phenomenex) combined with a SecurityGuard™ cartridge for HPLC Carbo-H columns with 3.2- to 8.0-mm internal diameters (4 × 3.0 mm, Phenomenex), at a controlled temperature of 50 °C. The mobile phase consisted of 100% water with 0.1% (v/v) formic acid, using an isocratic flow for 30 min. The flow rate was 250 µl min^−1^ and the injection volume was 2 µl.

The compounds were identified by ion trap technology and the mass spectrometric detection was realized with electron spray ionization (ESI; LTQ Velos, Thermo ScientificTM). Mass spectrometer (MS) spectra were recorded consecutively in one segment with two scan events in the range m/z 50.00–1000.00. In the first scan event, a full scan was conducted in a negative ion mode, and in the second scan, a full scan was realized in a positive ion mode. All data were evaluated and interpreted with Xcalibur and Mass FrontierTM Software (Thermo Scientific, Dreieich, Germany). The obtained data were identified by comparing the mass spectra and retention times with those of the reference compounds and characteristic fragmentation patterns using databases for substance classification or the mass spectra published in the literature. The reference compounds and LC–MS Chromasolv® grade water with 0.1% formic acid were obtained by Sigma-Aldrich, Switzerland. All of the standard samples were dissolved in a concentration of 1 mg ml^−1^ in pure water.

## Results

### Light microscopy and DNA staining

**Fig. 1:**
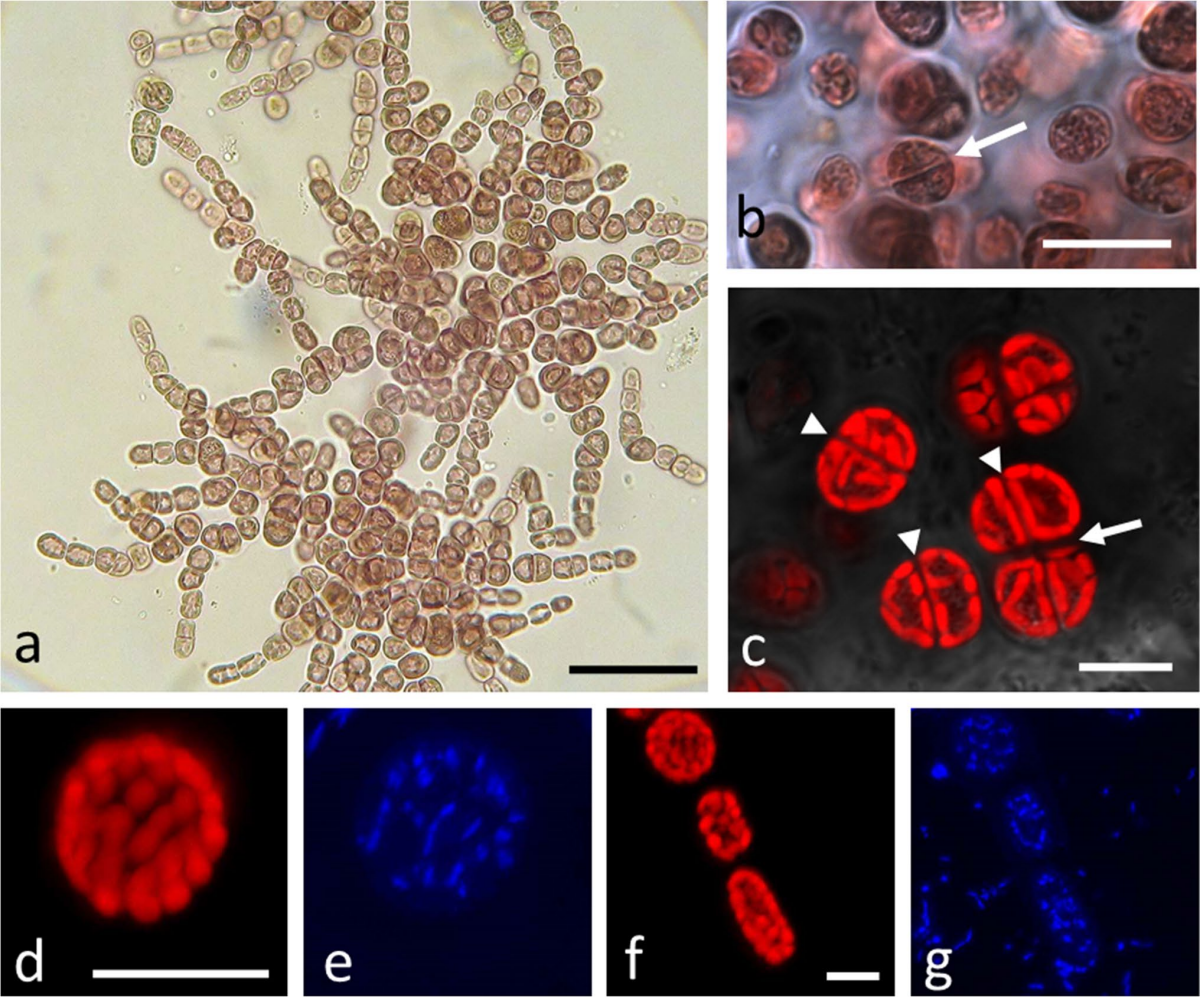
Light and confocal laser scanning microscopy (CLSM) of *Tsunamia transpacifica*. **a** Bright field image showing the typical cell architecture, **b** bright field image of cells grown at the University of Melbourne, detail with cells that have undergone cell division (arrow) grown at the University of Innsbruck, **c** CLSM image of the chlorophyll autofluorescence, transversal (arrow) and longitudinal (arrowhead) cell divisions, **c**, **d** chlorophyll autofluorescence of individual cell and **e** corresponding Hoechst stained image, **f** chlorophyll autofluorescence of accumulated cells, and **g** corresponding Hoechst stained image. Note that DNA staining is also visible outside the cells, presumably in the surrounding bacteria. Scale bars a 50 µm, b 20 µm, b–f 10 µm

*Tsunamia transpacifica* cells had the typical appearance of a well-developed crust with horizontal and vertical branching (Fig. 1a). Regularly cell divisions were observed indicating that the cultures were actively growing (Fig. 1b). By confocal laser scanning microscopy, transversal (Fig. 1c, arrow) and longitudinal (Fig. 1c, arrowhead) cell divisions were observed and the orientation of the parietal arranged chloroplasts was visible (Fig. 1c, d, f). The applied DNA staining protocols gave good signal for plastidal DNA (Fig. 1e, g), as the spots corresponded to the location of the chloroplasts, but did not stain the central nucleus. In Fig. 1f and g, a few cells embedded in an extracellular matrix are shown. DNA staining was evident in the chloroplasts, as well as on small spots outside the *T. transpacifica* cells, likely representing bacteria (Fig. 1g).

### Transmission electron microscopy shows thick cell walls, starch, and electron-dense bodies

**Fig. 2:**
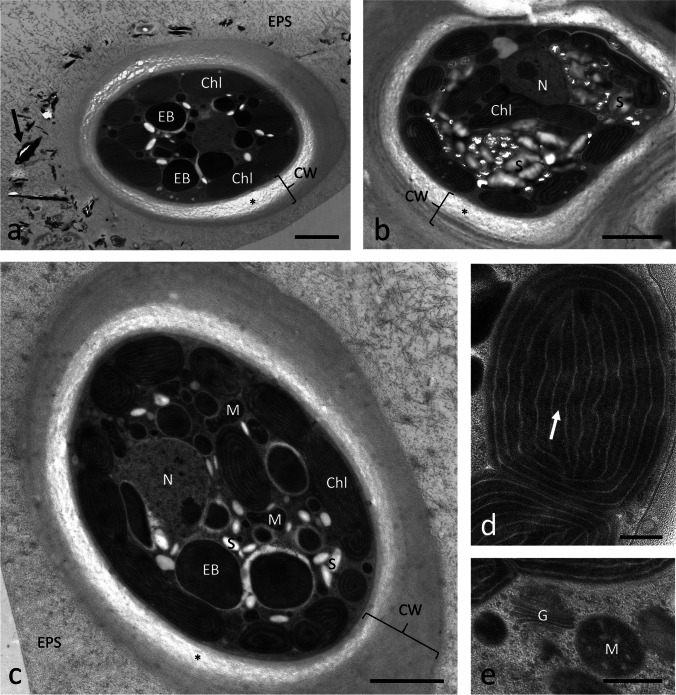
Transmission electron micrographs of HPF/FS *Tsunamia transpacifica* cells grown at the University of Innsbruck. **a** Individual cell surrounded by a cell wall which appears loose or not well infiltrated with the resin in the innermost layers (asterisk). The cell wall (CW) is surrounded by extracellular polymeric substances (EPS) containing electron-dense organic particles (arrow) and occasionally bacteria. The cytoplasm contains parietal chloroplasts, electron-dense bodies, **b** cell with numerous starch grains in the cytoplasm (= floridean starch), starch-free chloroplasts and a cell wall with an inner loose layer (asterisk), **c** cell wall with inner loose layer (asterisk) is surrounded by a distinct layer of EPS. The cell contains starch grains and mitochondria, chloroplasts are mostly arranged in the cell periphery, and electron-dense bodies are located in the inner cytoplasm and have a similar size as the chloroplasts but lack thylakoid membranes, **d** detail of individual chloroplast, arrow indicates electron-dense area between the electron translucent thylakoid membranes, **e** detail in the cell center with Golgi body and mitochondrion. Abbreviations: *Chl* chloroplast; *CW* cell wall; *EB* electron-dense body; *EPS* extracellular polymeric substances; *G* Golgi body; *N* nucleus; *M* mitochondrion. Scale bars a–c 2 µm, d–e 0.5 µm

**Fig. 3:**
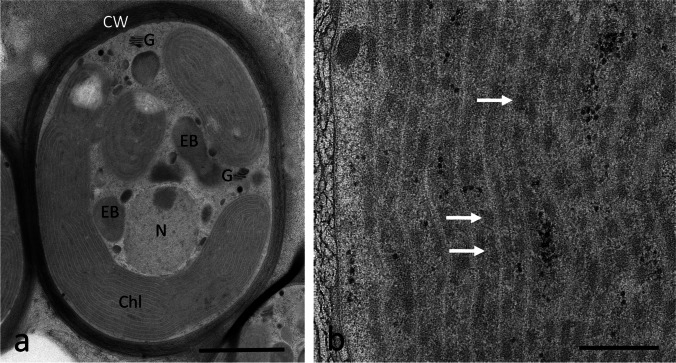
Transmission electron micrographs of HPF/FS *Tsunamia transpacifica* cells grown at the University of Melbourne. **a** Overview with parietal chloroplast (Chl), Golgi body (G), nucleus (N) electron-dense body (EB) appears small, cell wall (CW) is electron dense, **b** detail of the chloroplast, thylakoid membranes appear electron translucent and an electron-dense matrix was observed between the thylakoid membranes (arrows). Abbreviations: *Chl* chloroplast; *CW* cell wall; *EB* electron-dense body; *G* Golgi body; *N* nucleus; *M* mitochondrion. Scale bars a 2 µm, b 0.5 µm

High pressure freeze (HPF) fixation, followed by freeze substitution (FS), could sufficiently preserve the ultrastructure of *T. transpacifica* grown at the University of Innsbruck (Fig. 2) and at the University of Melbourne (Fig. 3), but chemical fixation failed (data not shown). *Tsunamia transpacifica* cells were surrounded by a thick cell wall (up to 2 µm); occasionally, the inner parts of the cell wall had a looser appearance (Fig. 2a). The individual cells with their distinct cell walls were embedded in a sheath of extracellular polymeric substances (EPS; Fig. 2a, c) that occasionally contained organic electron-dense particles or bacteria (Fig. 2a). The cellular ultrastructure allowed to depict the parietal chloroplasts (Fig. 2b–c). The chloroplasts had parallel-arranged thylakoid membranes, and the spaces between the thylakoid membranes contained an electron-dense matrix, but individual phycobilisomes could not be detected. Numerous electron-dense bodies were found in the cell center, with a diameter of up to 2–3 µm (Fig. 2a, c). Some cells contained numerous starch grains in the cytoplasm (Fig. 2b), but not in the chloroplasts. Mitochondria and Golgi bodies were well preserved (Fig. 2e) and the nucleus was located in the cell center (Fig. 2b, c). The specimens grown at the University of Melbourne showed a similar ultrastructure (Fig. 3a, b). Thylakoid membranes within the chloroplast were observed, but no clear phycobilisomes were detected (Fig. 3b).

### EELS measurements reveal an accumulation of phosphorus, cobalt, and iron

**Fig. 4:**
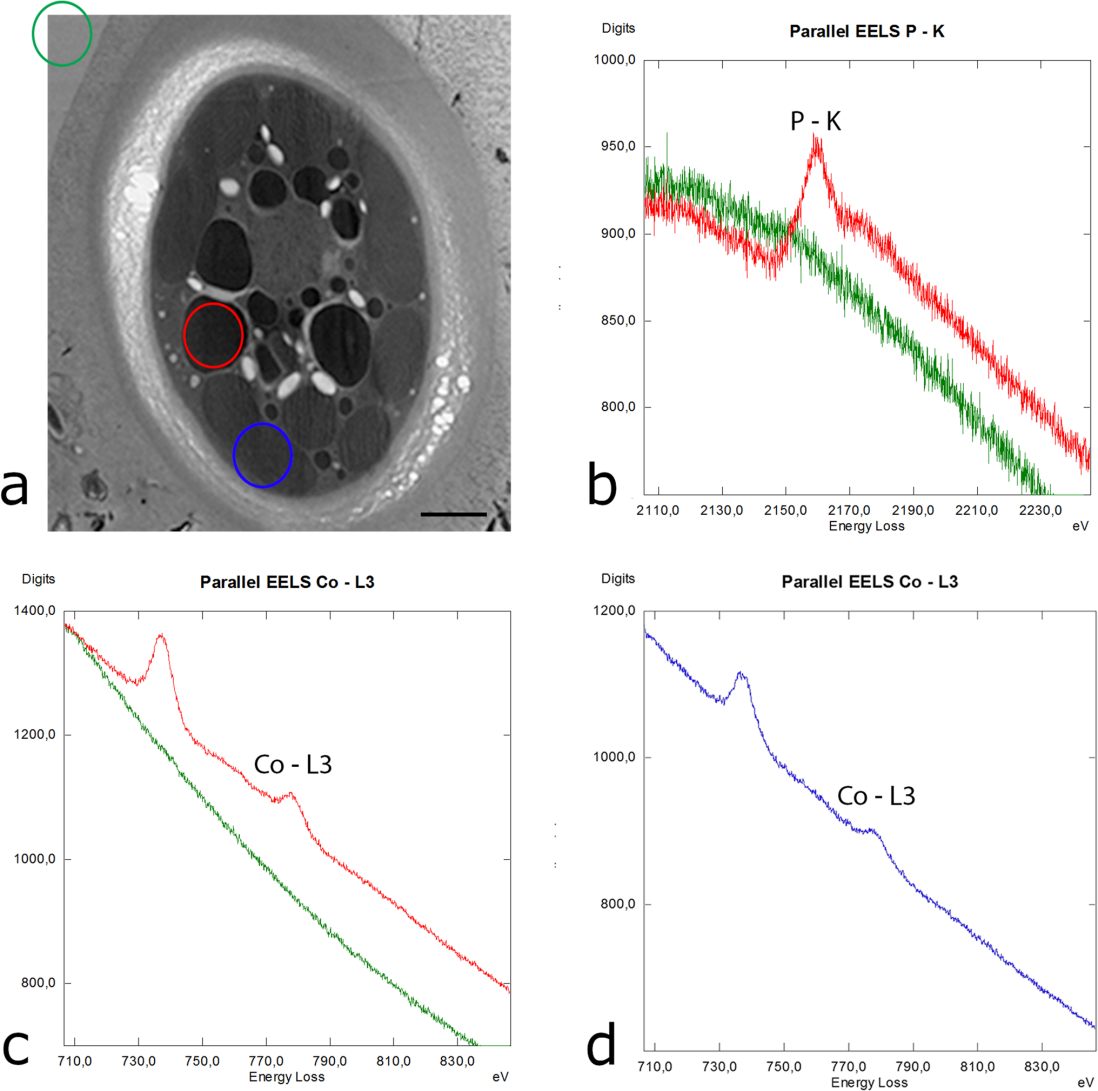
Electron energy loss spectroscopy (EELS) measurements in *Tsunamia transpacifica* grown at the University of Innsbruck. **a** Measurements of **b** phosphorus and **c**–**d** cobalt were performed in three different areas (red: electron-dense body, blue: chloroplast, green: resin outside the EPS) as indicated in the image in panel **a**. Scale bar 1 µm

**Fig. 5:**
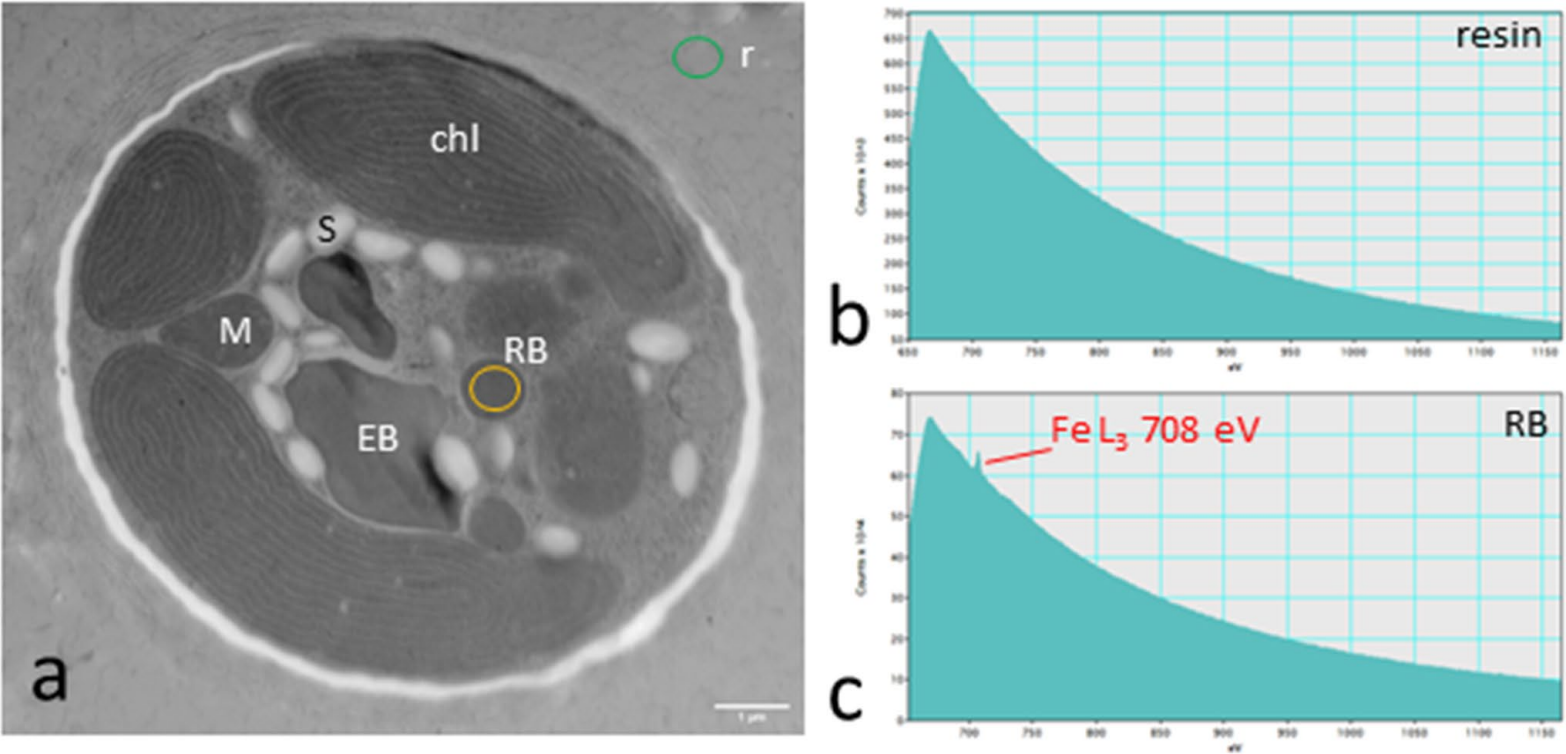
Electron energy loss spectroscopy (EELS) measurements in *Tsunamia transpacifica* grown at the University of Melbourne. **a** TEM overview with different areas of measurement indicated by circles, **b** EELS of resin outside the cell, **c** in rounded bodies (RB) iron was present, detected at the Fe L_3_ edge at 708 eV. Abbreviations: *chl* chloroplast; *M* mitochondrion, *EB* electron-dense body, *S* starch grain, *RB* rounded body. Scale bar = 1 µm

To further investigate the element composition within the cells and particularly the electron-dense bodies detected after HPF/FS (Fig. 2a, c), EELS measurements were performed in different areas of *T. transpacifica* cultured at the University of Innsbruck (Fig. 4a). These measurements revealed the occurrence of phosphorus (P) identified by the K-edge at 2146 eV in electron-dense bodies (Fig. 4b) and cobalt (Co) by the L3-edge at 779 eV in electron-dense bodies (Fig. 4c, red line), while a detection of these elements was missing outside the extracellular matrix of the cells (Fig. 4b, c; green line). Cobalt was also detected in chloroplasts of *T. transpacifica* (Fig. 4d, blue line). In *T. transpacifica* cultured at the University of Melbourne, additionally iron (Fe), determined by the L3-edge at 708 eV, was detected in smaller electron-dense rounded bodies (Fig. 5), whereas this element was not detectable in *T. transpacifica* cells cultured in Innsbruck.

### Detection of low molecular weight carbohydrates suggests osmotic protection

**Fig. 6:**
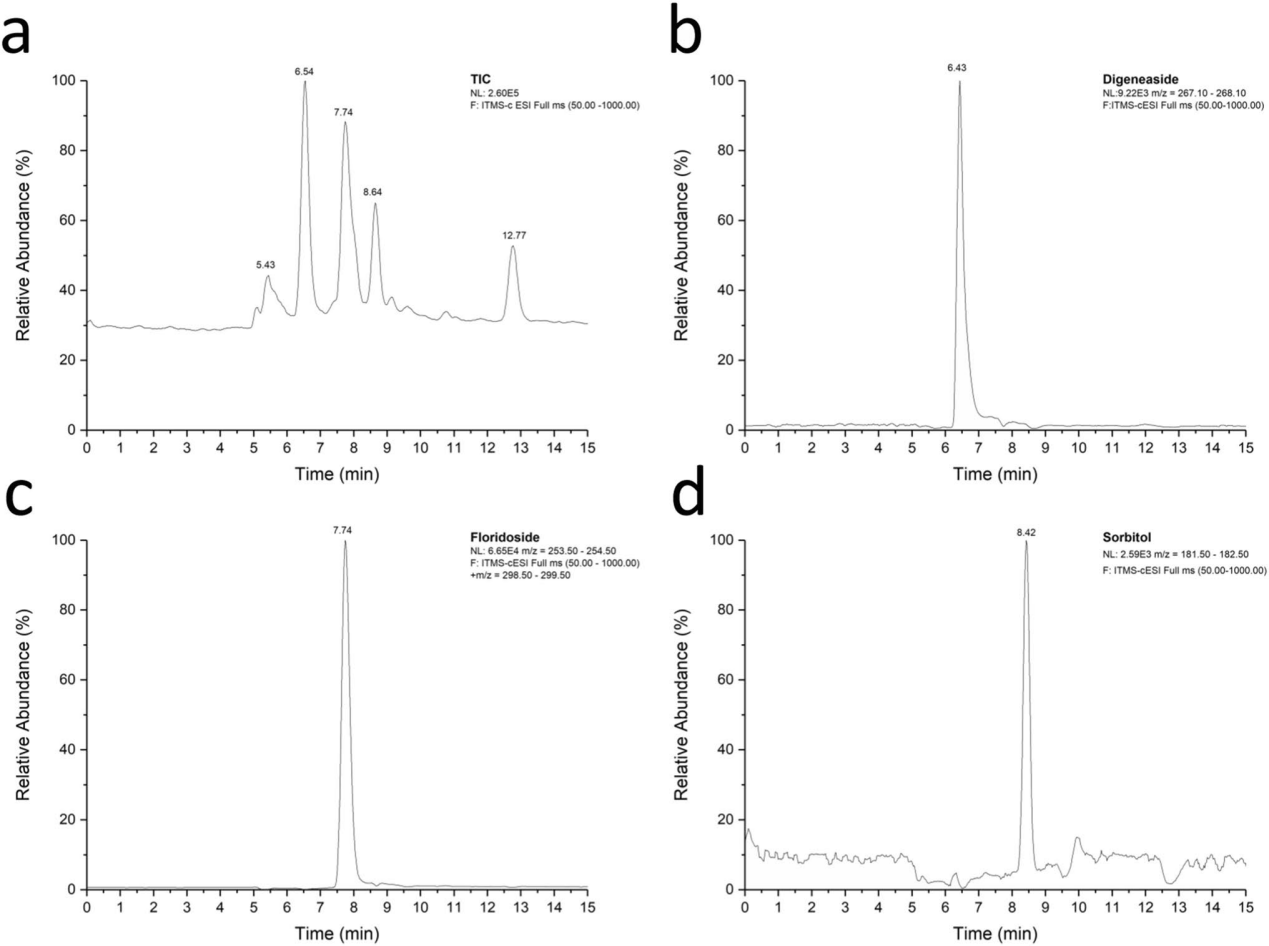
Liquid chromatography (LC) of an aqueous sample of *Tsunamia transpacifica*. Shown is the relative abundance of the major low molecular weight carbohydrate peaks. **a** Total ion current (TIC). Three peaks could be identified as **b** digeneaside, **c** floridoside, and **d** sorbitol

**Fig. 7:**
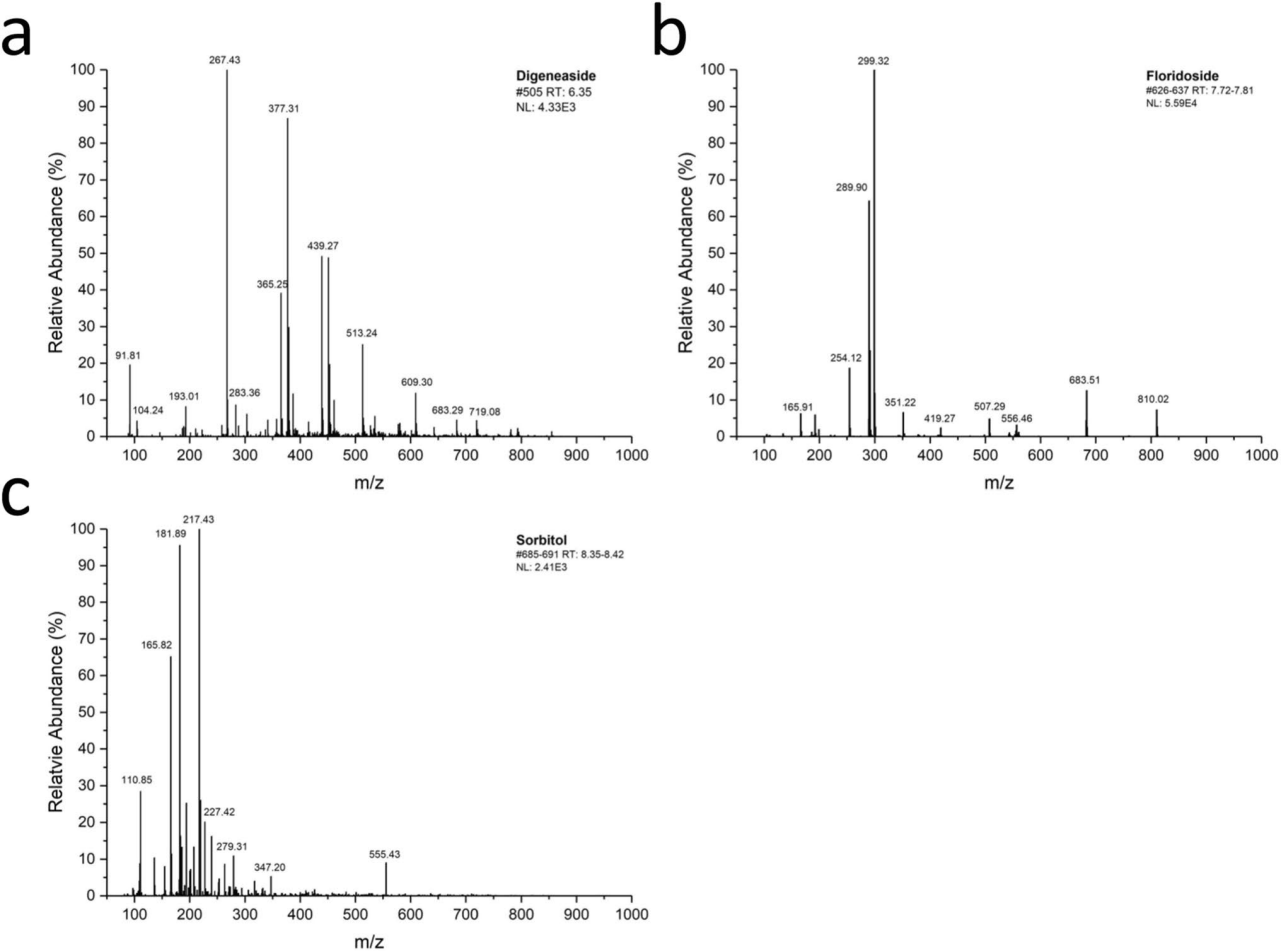
Electron spray ionization (ESI)-MS spectra of the identified low molecular weight carbohydrates. **a** Digeneaside, **b** floridoside, and **c** sorbitol

The HPLC and LC–MS data clearly indicate the presence of the heterosides digeneaside and floridoside together with sorbitol as main LMWCs in cell extracts of *T. transpacifica* (Figs. 6, 7). In addition, traces of about 10 other carbohydrates were observed, for example, conspicuous peaks at 5.43 and 12.77 min, some minor peaks in between (Fig. 6a), as well as small peaks > 15 min (not shown), but their chemical identity could not be determined. The digeneaside concentration was 14.4 ± 3.7 µmol g^−1^ DW, that of floridoside 5 times higher (70.5 ± 9.8 µmol g^−1^ DW) and sorbitol accounted for 121.6 ± 8.7 µmol g^−1^ DW (each concentration based on 3 replicates).

Standard solutions and samples were analyzed by using ESI–MS. The detection of LMWCs was possible in both ion modes, but the signal intensity was much better in the negative ESI mode. Figure 6a shows the total ion current (TIC) for the algal sample. The three highest peaks reveal the investigated LMWCs in the total run of the *T. transpacifica* sample (Fig. 6a). It was possible to detect five different peaks. The three highest peaks represent the investigated LMWCs in the total run of the algal sample. After comparison with standard measurements, three LMWCs could be definitely identified. Their isolated chromatograms from the TIC are also shown in Fig. 6b–d: digeneaside with a retention time of 6.43 min (Fig. 6b), followed by floridoside (Fig. 6c) with a retention time of 7.74 min, and sorbitol at 8.42 min (Fig. 6d).

The ESI–MS spectra of the identified LMWCs are given with their characteristic fingerprint profile (Fig. 7a–c). For floridoside (Fig. 7b), in addition to the precursor ion [M-H]- with m/z = 254, the adduct with formic acid [M + Fac]- with m/z = 299 and further target fragments with m/z 289 and m/z 165 are also shown. The MS profiles of sorbitol (Fig. 7c) and digeneaside (Fig. 7a) present the precursor ions [M-H]- with m/z = 181 along with m/z 217, m/z 227, and m/z 279 for sorbitol and m/z = 267 along with m/z 289 and m/z 193 for digeneaside. Therefore, these mass spectroscopic data confirm the presence of the three LMWCs in *T. transpacifica.*

## Discussion

In the present study, we provide a comprehensive structural and ultrastructural description, EELS element analysis, and a qualitative and quantitative determination of the soluble LMWCs of *T. transpacifica*. These are the first results on properties of the recently described genus *Tsunamia* (West et al. 2016). These data will help to understand the rather unique ecology of this alga. *Tsunamia transpacifica* has the astonishing ability to thrive for a long time on the ocean’s surface and is able to survive when drifted ashore. Under these differing conditions, particularly solar radiation and irregular dehydration are two of the main stressors which the alga can cope with.

Our TEM data confirmed the light microscopical observations on the parietal chloroplasts without pyrenoids (West et al. 2016). The chloroplast lobes showed parallel-arranged thylakoid membranes, typical for red algae and with a similar appearance as in other members of the Stylonematophyceae (e.g., West et al. 2005, 2007). In contrast, the large chloroplast of *Rhodaphanes brevistipitata* showed a central pyrenoid (West et al. 2007). The chloroplast of *Rhodaphanes* did not show an encircling thylakoid which is in contrast to *T. transpacifica* where up to three encircling thylakoids were observed. Distinct phycobilisomes were difficult to spot in *T. transpacifica* after the HPF/FS protocols applied in this study. The contents of the plastids and spaces between the thylakoid membranes appeared electron dense, suggesting a good structural preservation. The cytoplasm of several cells of *T. transpacifica* contained large amounts of starch grains, accounting for high physiological activity synthesizing this storage compound as was also observed in other red algae like *Batrachospermum turfosum* (Aigner et al. 2017). It was surprising to get absolutely non-satisfying results after chemical fixation for preserving the ultrastructure of *T. transpacifica*, a technique that is well established and has provided reasonable results in several other red algae such as *Palmaria palmata* and *Odonthalia dentata* (Holzinger et al. 2004) and the freshwater red alga *B. turfosum* (Aigner et al. 2017) as some examples from our laboratory.

Initially, we performed DNA staining to visualize the cell’s nuclei. However, we saw that common DNA dyes, like Hoechst 33,342 (Fig. 1e, g) and SYBR Green (not shown), only stained plastidal DNA in *T. transpacifica*, but were excluded from the nucleus. We conclude that the nuclear envelope of *T. transpacifica* is not permanent for these dyes, despite different fixations and staining procedures have been employed that were successful in nuclear staining in *Viator vitreocola* (Hansen et al. 2019). At the present stage, we cannot give an explanation which components of the nuclear envelope are responsible for this exclusion, so further examination is needed.

### Ultrastructure and properties of Tsunamia cell walls

The cells were surrounded by a thick, multilayered cell wall, up to 2 µm in diameter. Currently, we do not have detailed information on the biochemical composition of the cell wall and the extracellular matrix of *T. transpacifica*, but the respective analysis is on the way, and there are some indications that xyloglucans and arabinogalactan proteins could play an important role in the adhesion mechanisms (Veenhof and West 2018; Zoe Popper, Galway, personal communication). In addition, *Chroodactylon ornatum*, also a member of the Stylonematophyceae, has been described to contain sulfated galactans in their mucilaginous sheath (Cabrera et al. 2014). Most importantly, we provide here ultrastructural evidence that in *T. transpacifica*, several cells covered by distinct cell walls are embedded together in an extracellular matrix composed of extracellular polymeric substances (EPS), which appears denser towards the outside. These EPS may contain organic electron-dense particles or bacteria, and their occurrence is likely the reason why these cells have the ability to stick tightly to the plastic surfaces. A possible factor for the difficulties in fixation of this alga is likely the chemical composition of the cell walls and the EPS, which possibly prevent proper resin infiltration.

### Electron-dense bodies likely represent chloroplast-derived structures

Prominent electron-dense bodies similar in size and appearance as chloroplasts but lacking their internal structure with thylakoids were found in *T. transpacifica* samples. These bodies were found in different size classes, most of them in the dimensions of a chloroplast (i.e., ~ 1–2 µm), but also smaller “rounded bodies,” which may have a different content. The interpretation of these structures is difficult, but at least the smaller ones could resemble the brownish granules detected by LM in a previous study (West et al. (2016), Fig. 1c, cell indicated with arrow).

While electron-dense bodies are frequently observed in brown algae, where they are described as physodes, for example, in the Arctic kelp *Saccharina latissima* (Holzinger et al. 2011), they are uncommon for red algae. Physodes are described as phlorotannin-containing vesicles and due to their spectral properties have well characterized protective functions against enhanced UV radiation (Shibata et al. 2004; Schoenwaelder 2002). However, it seemed unlikely that the electron-dense bodies in *T. transpacifica* have a similar content, and the records of phlorotannins in red algae are sparse.

Thus, we speculate that the electron-dense bodies are possibly chloroplast-derived and could resemble degradation products of the chloroplasts. This is supported by the size of these structures and their inhomogeneous contents. An interesting observation supporting this interpretation of the electron-dense bodies was made by Coelho et al. (1998) who investigated a marine snail that produced its purple defensive ink exclusively from the accessory photosynthetic pigment phycoerythrin, deriving from the red seaweeds these snails are consuming. Interestingly, these authors showed TEM micrographs of “electron-dense granules” in the digestive vacuoles of red algae-fed snails which remarkably resemble our observations of the electron-dense bodies. By means of immunolabelling, Coelho et al. (1998) provided evidence for the chemical nature of the osmiophilic material and were able to detect P, S, Cl, Cr, and Fe by means of X-ray spectrum analysis (EDS) within a rhodoplast digestive vacuole. These data are partially similar to our observations; however, we did not detect Cr, but Co instead. Moreover, it should be tested if the electron-dense bodies in *T. transpacifica* also derived from phycoerythrin degradation.

### Phosphorus, cobalt, and iron are accumulated in electron-dense bodies of Tsunamia

Phosphorus is ubiquitously present in the marine water column due to biological activity, weathering of P-rich sediments, and coastal upwelling, as well as from anthropogenic inputs into coastal waters such as fertilizers (White and Dyhrman 2013). The situation might be different in the open ocean where *T. transpacifica* was drifting and P can be temporarily limiting in certain situations in the North Pacific (Conkright et al. 2000). Some red algae show a bi-phasic kinetics of P uptake with saturable kinetics at low concentrations and a linear uptake at high external P concentrations (Friedlander and Dawes 1985). In the red alga *Palmaria palmata*, P uptake was bi-phasic without saturation (Martinez and Rico 2004). The biological function of P accumulation in red algae is likely the storage of this nutrient for periods of P deficiency.

A “luxury” consumption of P in freshwater benthic algae has been described by Stevenson and Stoermer (1982). These authors stated that the abilities of different algae to absorb nutrients are important ecological traits for the interspecific competition, and they found the second highest accumulation of polyphosphate bodies in *Asterocytis smaragdina* (Bangiophyceae) when compared to algal epiphytes from other phylogenetic positions like *Cocconeis pediculus* (diatom), which exhibited the highest density of polyphosphate bodies (Stevenson and Stoermer 1982). The number and size of polyphosphate granules is a suitable cellular indicator for eutrophication, i.e. the amount of phosphate in surface waters (Eixler et al. 2006). These authors also performed different localization experiments in the cyanobacterium *Synechocystis* sp. and the green alga *Chlorella vulgaris* (Eixler et al. 2005). By means of electron spectroscopic imaging, they found the most electron-dense (dark) areas that were corresponding to the highest P concentrations (Eixler et al. 2005). Also, Ota et al. (2016) draw a clear relationship between polyphosphate bodies and the accumulation of electron-dense bodies in the green alga *Parachlorella kessleri*. In this species, sulfur-depleted conditions led to the accumulation of P, but the respective signal was only detected in electron-dense bodies by energy dispersive X-ray analysis (Ota et al. 2016). In *T. transpacifica*, an even more sophisticated method, EELS, was employed that clearly detected accumulation of P in electron-dense bodies. When viewing electron-dense bodies from a structural perspective, also similarities to polyphosphate bodies described in cyanobacteria of the early TEM literature (Jensen 1968; Jensen and Sicko 1974; Jensen et al. 1977).

An accumulation of Co has been observed in electron-dense bodies as well as in intact chloroplasts in *Tsunamia* (Fig. 4d, f), further supporting the speculation that electron-dense bodies are derived from chloroplasts. Cobalt (II) is usually highly toxic to organisms and in particular to the photosynthetic apparatus of some algae and may be deposited there (e.g., El-Sheekh et al. 2003; Macfie et al. 1994). Thus, sequestering this heavy metal is likely beneficial for the alga and might in turn lead to changed and degraded chloroplasts. In contrast, an accumulation of Fe was only found in small rounded electron-dense bodies of *Tsunamia* grown in Melbourne.

However, at this stage, we can only speculate on the interpretation of these data. An effect of the cultivation medium is likely to be excluded as both media have basically the same chemical composition. However, light and temperature conditions for cultivation in the laboratories were different (5- to 10-µmol photons m^−2^ s^−1^ at 18–22 °C *versus* 6- to 30-µmol photons m^−2^ s^−1^ at 14–20 °C), and particularly the higher photosynthetic active radiation (PAR) in combination with lower temperatures could explain the contrasting results of *Tsunamia* grown at the University of Melbourne and at the University of Innsbruck.

Red algae such as *Hypnea valentiae* have the capacity to remove Co (II) ions from aqueous solutions in a pH- and temperature-dependent manner (Vafajoo et al. 2017). The biosorption of Co (II) has been tested in various seaweeds including the red algae *Gracilaria edulis* where the in situ uptake capacities were in the medium range and highest at pH 4–5 (Vijayaraghavana et al. 2005). Bioaccumulation of Co has been described in several red algae including *Porphyra tenera*, *Palmaria palmata*, *Chondrus crispus* (Kuyucak and Volesky 1988a), and *Corallina elongata* (Benabdallah et al. 2018). The earlier authors described the mechanism of biosorption of Co (II) in the brown alga *Ascophyllum nodosum* by physical binding to cell walls (Kuyucak and Volesky 1988b). The biosorption mechanisms involved are predominately ion exchange by the sulfated alginates of the cell wall which play a crucial role in the Co (II) binding whereas transport/passage of Co (II) across the cell wall/plasmalemma into the cell occurs to a lower rate and possibly in a temperature-dependent manner. However, these studies do not give any information on where the sequestered metal ions are localized. Indications for Fe (II) bioavailability in red algae are evident from the literature, and concentrations of 196 mg 100 g^−1^ dry weight were found in *Gracilariopsis* (Garcia-Casal et al. 2008) and *Porphyra* (Garcia-Casal et al. 2009). The accumulation of Fe (II) in small round electron-dense bodies in cells grown at the University of Melbourne might reflect the biosorption capacities of *T. transpacifica* for this trace element, whereas EELS of cells grown at the University of Innsbruck did not allow to detect Fe (II). Usually, Fe (II) is poorly soluble in sea water, and hence often limiting to phytoplankton growth as it is necessary for photosynthesis.

### Putative function of soluble LMWCs as protective compounds

Marine algae that are frequently exposed to salinity changes or desiccation typically follow the metabolic strategy to keep the sodium and chloride concentrations in the cytoplasm as low as possible, because both ions negatively affect protein and organelle function, membrane integrity, and structural macromolecules (Kirst 1990). Instead and to generate sufficient osmotic pressure, organic osmolytes are synthesized and accumulated in the cytoplasm, which are compatible to all metabolic functions (Eggert and Karsten 2010). In red algae, numerous LMWCs have been identified (Eggert and Karsten 2010), and these often represent the main photosynthetic product. Most orders of red algae synthesize the heteroside floridoside, while most members of the Ceramiales generally form and accumulate instead of floridoside, the chemically related digeneaside (Kremer 1978). Most interesting is the observation that in some Ceramiales such as in the mangrove-associated genera *Bostrychia* and *Stictosiphonia* as well as in some early diverging red algal lineages (e.g., Stylonematophyceae), the polyol sorbitol can be found (Eggert and Karsten 2010), which is otherwise uncommon for red algae. With the exception of digeneaside that plays no more than a minor role in osmotic acclimation of red algae (Eggert and Karsten 2010), floridoside and sorbitol act as organic osmolytes. Our data clearly indicate that digeneaside, floridoside, and sorbitol occur together in *Tsunamia*, but in different concentrations of 14.4, 70.5, and 121.6 µmol g^−1^ DW, respectively. These amounts already reflect their osmotic relevance, i.e. the higher the content the more the compound plays a significant role in osmotic acclimation (Kirst 1990). Since these organic compounds can be accumulated and tolerated at high intracellular concentrations, and permit the generation of low water potentials without incurring metabolic damage (Yancey 2005), the term “compatible solute” was introduced by Brown and Simpson (1972). In general, the intracellular concentrations of floridoside and sorbitol are actively adjusted by photosynthesis-driven de novo biosynthesis or by remobilization of storage products and are directly proportional to external salinity or desiccation (Kirst 1990). In addition, the function of sorbitol and floridoside as compatible solutes was experimentally proven on enzyme extracts that originated from various mangrove red algae (Karsten et al. 1996). The in vitro activity of two key enzymes was strongly inhibited with increasing NaCl concentrations, while equimolar concentrations of sorbitol or floridoside did not inhibit enzyme function, with sorbitol exhibiting even a slightly stimulating effect (Karsten et al. 1996). In contrast, a comprehensive biochemical study on digeneaside in a euryhaline red alga experimentally proved that the content of this heteroside always remained low and unaffected by salinity (Karsten et al. 1994), and hence is not involved in osmotic acclimation.

So far, the joint occurrence of floridoside and digeneaside in red algae was reported in *Stylonema alsidii* and *Stylonema cornu-cervi* (Karsten et al. 1999) and later in *Rhodochaete parvula* (Karsten et al. 2003), which are all considered taxa of early diverging lineages (Stylonematophyceae, Compsopogonophyceae). In addition, in *S. alsidii* and *S. cornu-cervi*, sorbitol occurred as third LMWC (Karsten et al. 1999). However, also in some lineages of the Porphyridiophyceae (*Erythrolobus coxiae*, *E. madagascariensis*, and *Timspurckia multipyrenoidosa*), the co-occurrence of these two heterosides was reported, while members of *Porphyridium* and *Flintiella* possess only floridoside (Yang et al. 2010). The distribution patterns of these LMWCs in early diverging lineages of the Rhodophyta are of chemotaxonomic value (Karsten et al. 1999).

## Conclusion

The here presented data increase our knowledge on the ultrastructure and element composition in Stylonematophyceae, presenting novel TEM and EELS data. Our hypothesis on storage and protection mechanisms could be confirmed with the presented data. An accumulation of P and Co or Fe was found in electron-dense bodies, presumably resembling degraded chloroplasts. It should be further tested if these structures contain degradation products of phycoerythrin (e.g., Coelho et al. 1998, González-Ramírez et al. 2014). The biosorption capacities for P and trace elements might be beneficial for *T. transpacifica* drifting for extended times in nutrient-poor surface waters. The occurrence and composition of LMWCs in *T. transpacifica* matches well with its phylogenetic position in Stylonematophyceae. Particularly, floridoside and sorbitol act as compatible solutes and allow survival upon desiccation events expected when the plastic particles are drifted ashore. The cells encompass very thick cell walls and are embedded in EPS, likely responsible for the adhering capacities of this alga supporting our third hypothesis. Future investigations should elucidate adhesion mechanisms and the chemical composition of the cell walls. Experimentally testing the strength of adhesion to different artificial substrata would further elucidate our understanding of red algae “naturally” growing on ocean debris. These organisms have preferences for certain substrata like plastic or glass (Hansen et al. 2019), with different surface properties. Of course, an elucidation of the original natural habitat would be interesting; however, as these organisms are small and inconspicuous, this is a difficult task.

## Abbreviations

EELS: Electron energy loss spectroscopy
ESI: Electron spray ionization
EPS: Extracellular polymeric substances
LC-MS: Liquid chromatography-mass spectrometry
LMWC: Low molecular weight carbohydrate
MPM: Modified Provasoli’s medium
MSS: Morton sea salt
NSW: Natural sea water
TIC: Total ion current
